# WFS1-related isolated diabetes induced by a WFS1 missense mutation: focus on the isolated diabetes phenotype

**DOI:** 10.1186/s13023-026-04291-9

**Published:** 2026-03-27

**Authors:** Mei Huang, Qianying Wei, Yao Qin, Lu Qin, Ying Shao, Suyu Wang, Xinyi Qu, Tao Yang, Mei Zhang

**Affiliations:** Department of Endocrinology, The First Affiliated Hospital with Nanjing Medical University, Nanjing Medical University, 300 Guangzhou Road, Nanjing, Jiangsu 210029 P.R. China

**Keywords:** WFS1 gene, Beta-cell function, Non-syndromic phenotype, Isolated diabetes

## Abstract

**Background:**

Wolfram syndrome is a rare disease caused by the mutation of WFS1 gene, characterized as s spectrum of disorders. We aim to investigate the clinical features and pathogenic mechanisms of a WFS1 missense mutation inducing atypical phenotype characterized solely by isolated diabetes mellitus (DM).

**Results:**

Proband and her extended families were investigated to identify mutations in the WFS1 gene. Islet function analysis was conducted to assess beta-cell function of the proband and other mutation carriers and the proband was follow-up for five years. HOMA-IR and HOMA-β were calculated to reflect insulin sensitivity and beta-cell function. In vitro experiments were conducted to evaluate the effects of WFS1 mutations. Through transfection of wild type (WT) and mutant WFS1 plasmids into MIN6 cells, we investigate the effects of mutations on endoplasmic reticulum (ER) stress and insulin processing. A missense mutation in WFS1 (c.173 C > T, p.A58V) was identified in a patient with non-syndromic diabetes and five of her family members. Islet function analysis suggested heterogeneity of beta-cell function, with notable differences of HOMA-IR and HOMA-β among the proband and family members. In vitro experiments demonstrated that the WFS1 mutations activates ER stress response, leading to an increase in the levels of p-PERK, XBP1s, ATF4 and pIRE1-α. WFS1 mutations also led to impaired insulin biosynthesis, manifested by the accumulation of proinsulin and an increased proinsulin-to-insulin ratio.

**Conclusion:**

The p.A58V WFS1 mutation is implicated in a non-syndromic DM phenotype and variable beta-cell function. This mutation activates ER stress pathways and disrupts proinsulin processing, offering insights into the molecular pathogenesis of this unique form of WS.

**Supplementary Information:**

The online version contains supplementary material available at 10.1186/s13023-026-04291-9.

## Background

Wolfram syndrome (WS) is a rare neurodegenerative disorder with a broad phenotypic spectrum of classical syndromes, including diabetes mellitus (DM) and optic atrophy (OA) which is often accompanied by diabetes insipidus (DI), deafness, urological and neurological complications in combination or in isolation [1]. Classical WS was first described in 1938 by Wolfram and Wagener [2], with a prevalence of approximately 1/160,000 to 1/77,000 [3]. However, less than 55% reported cases have the complete phenotype, growing evidence revealed atypical conditions of non-syndromic diabetes phenotype, notably isolated DM, which can be misdiagnosed of type 1 diabetes mellitus (T1DM) due to similarities [4]. According to previous reports, a significant prevalence of WS (6%) was identified in T1DM population [5]. This data is in accordance with the suggestion that confounding diagnosis of T1DM and atypical WS may result in underestimated incidence of WS and a low vigilance of medical staff toward the disease.

The pathogenesis of WS is linked to mutations in the WFS1 gene, situated on chromosome 4p 16.1. The WFS1 gene encodes Wolframin, an 890 amino acid transmembrane protein [6], playing a critical role in regulating endoplasmic reticulum (ER) calcium homeostasis and unfolded protein responses (UPR) [7]. Wolframin is involved in multiple organs, including pancreas, brain, kidney and liver, playing important role in maintaining physiological functions. Wolframin is especially highly expressed in the pancreatic beta cells, participating in insulin biosynthesis and insulin production, which is critical for normal glucose regulation [8].

Wolframin’s dysfunction, attributable to its pivotal role in ER homeostasis, precipitates ER stress in the highly susceptible secretory pancreatic beta cells, leading to aberrant insulin synthesis and secretion [9]. Additionally, WFS1’s localization in secretory granules within beta cells suggests its involvement in insulin processing through intragranular acidification, a process that, when impaired, affects beta cell function [10]. Despite these insights, research directly assessing beta-cell function in WS patients remains scarce. Meanwhile, as pathogenic variants of WFS1, there are marked variability in age of onset, progression, and severity of symptoms. Family herterogeneity is critical to unraveling the clinical and mechanistic complexity of disease, indicating the influence of genetic modifiers, environmental factors, or epigenetic mechanisms. Exploring changes in pancreatic islet function helps clarify the unrecognized pathways that affect beta-cell survival and dysfunction, and can also promote personalized therapeutic strategies.

In the present study, we have identified a missense mutation in the WFS1 gene in a patient with an atypical phenotype characterized solely by DM and revealed a spectrum of beta-cell function heterogeneity within her family pedigree. To further investigate the mutation’s deleterious impact on beta-cell functionality associated with this phenotype, we conducted in vitro experiments to evaluate the functional impact of this identified variant. Our research aims to contribute novel insights into the diverse clinical presentations of WFS1 gene and to elucidate its underlying pathogenic mechanisms of DM.

## Methods

### Patient characteristics and beta-cell function evaluation

The proband was a 14-year-old girl, who was hospitalized in the First Affiliated Hospital with Nanjing Medical University, presenting primarily with one-year-long history of polydipsia. Meanwhile, the proband reported no concomitant vision or auditory impairment. A familial predisposition to diabetes was noted, with both her mother and maternal grandmother having a history of the condition. Comprehensive evaluations including physical examinations, laboratory diagnostics, and instrumental assessments were conducted on the patient. Family members were included if they were direct relatives of the proband and voluntarily agreed to participate. Informed consents were obtained from the patient and her family members according to a protocol approved by the Ethics Committee of the First Affiliated Hospital with Nanjing Medical University (2019-SR-121.A1) and in compliance with the Declaration of Helsinki.

The assessment of beta-cell function was conducted using an Oral Glucose Tolerance Test (OGTT). After a 10- to 12-h overnight fast, subjects consumed a 75-g glucose within 5 min. Blood samples were taken at 0, 60 and 120 min for the measurement of glucose, insulin and C-peptide concentrations. Serum glucose was measured with an automatic enzymatic analyzer (Beckman Coulter, USA). Serum C-peptide and insulin levels were measured by a chemiluminescence assay (Roche Diagnostics, Switzerland). Indices include homeostasis model assessment of insulin resistance (HOMA-IR) and HOMA-β are used to further reflect insulin sensitivity and pancreatic beta-cell function. The calculation formula of HOMA-IR is as follows: Fasting Insulin (mU/L)×Fasting Glucose (mmol/L)/22.5; the calculation formula of HOMA-β is as follows: 20×Fasting Insulin (mU/L)/(Fasting Glucose (mmol/L)-3.5).

### WFS1 gene sequencing and genetic analysis

For mutation analysis, blood samples were procured from the patient, along with her immediate family members, including parents, maternal grandparents, maternal aunt and cousin. Genomic DNA was extracted from the peripheral blood samples using the QIAamp DNA Mini Kit (Qiagen, Shanghai, China). Primers were designed targeting exons and the exon–intron sequences of WFS1. Then, the genes were amplified by PCR, the products were sent for Sanger sequencing to validate the identified mutation using specific primers.

We applied a series of computational programs to comprehensively predict the pathogenicity of the candidate variant, including REVEL (https://sites.google.com/site/revelgenomics/), PolyPhen_2 (http://genetics.bwh.harvard.edu/pph2/), Mutation Taster (http://www.mutationtaster.org/), and GERP (http://mendel.stanford.edu/SidowLab/downloads/gerp/).

We further built a three-dimensional (3D) model of the Wolframin protein by Alphafold2 and presented the wild-type and mutant protein structures to demonstrate the discrepancy in structure. And we also created a figure to demonstrate the transmembrane protein Wolframin. The rendered images were generated using PyMOL (v2.5).

### Plasmids construction, cell culture and transfection

The wild type (WT) WFS1 plasmid was constructed by inserting the full-length of the WFS1 sequence (GenBank: NM_006005.3) along with a flag tag sequence into the pcDNA3.1-T2A-EGFP vector. While the p.A58V mutant plasmid was constructed by incorporating the specific mutation of c.173 C > T. Additionally, the 425ins16 mutant plasmid was generated by introducing a 16 bp-insertion (GGCCGTCGCGAGGCTG) insertion into the WT WFS1 plasmid according to the protocols provided by the Thermo Scientific Phusion kit (Thermo Scientific). The mutation 425ins16 was reported in a Spanish degree, presenting a full spectrum of 4 classical syndromes, serving as a control to p.A58V mutation [11].

MIN6 cells were cultured in Dulbecco’s modification of Eagle’s medium (DMEM; 25mmol/L glucose) supplemented with 15% fetal bovine serum (FBS), 1% penicillin-streptomycin and 50 µM β-mercaptoethanol under standard conditions (5% CO_2_ and 37℃). MIN6 cells were transiently transfected with pcDNA3.1(+)-Flag, wild type, p.A58V, 425ins16 WFS1 expression plasmid using the RFect plasmid DNA transfection kit, following the manufacturer’s guidelines. Post-transfection, the cells were cultured for 24 h to prepare for subsequent assays.

### Quantitative real-time PCR

The day before transfection, MIN6 cells were plated on a 12-well plate at a density of 4 × 10^5^/well. Twenty-four hours after transfection, total RNA was extracted using Trizol reagent (Takara), followed by reverse transcription into cDNA using the HiScript^®^ III All-in-one RT SuperMix Kit (Vazyme). Real-time quantitative PCR analysis was performed using ChamQ SYBR Green supermix (Vazyme) on a StepOnePlus qRT-PCR detection system (Thermo Fisher Scientific). Primer sequences synthesized by Invitrogen Biotechnology (Shanghai, China) are detailed in Supplementary Table 1. All experiments were performed in triplicate, and the data were calculated as the fold-change using the 2 − ΔΔCt method. Expression levels of the target gene were normalized to the internal reference gene β-actin.

### Western blotting

MIN6 cells were collected and lysed by radio immunoprecipitation assay (RIPA) lysis buffer, supplemented with protease inhibitor and phosphatase inhibitor (MedChemExpress). The concentration of total protein was determined via the bicinchoninic acid (BCA) assay (Beyotime). The denatured protein was separated by 10% sodium dodecyl sulfate polyacrylamide gel electrophoresis (SDS-PAGE), and transferred to polyvinylidene fluoride (PVDF) membranes with a pore size of 0.45 μm (Millipore). Membranes were subsequently blocked with 5% bovine serum albumin, and then probed overnight at 4 °C with primary anti-WFS1 (26995-1-AP, Proteintech 1:1,000), anti-IRE1-α (HY-P80828, MCE; 1:1,000), anti-pIRE1-α (HY-P86330; MCE; 1:1000) anti-PERK (HY-P80781, MCE; 1:1,000), anti-pPERK (HY-P81190; MCE; 1:1000); anti-ATF4(HY-P80486, MCE; 1:1,000); and anti-actin (66009-1-Ig, Proteintech; 1:2,0000) antibodies, followed by 1.5 h incubation at room temperature with secondary horseradish peroxidase–conjugated antibody (goat anti-rabbit, 7074P2, CST; 1:3,000 or goat anti-mouse, A0216, Beyotime; 1:1,000). Protein bands were detected by ChemiDoc XRS+ Chemiluminescence imaging system (BioRad). β-actin served as a loading control. The grey value of each band was analyzed using Image J software.

### Immunofluorescence staining

MIN6 cells were grown on glass coverslips for 24 h, washed with phosphate-buffered saline (PBS) and fixed with 4% paraformaldehyde (Biosharp) for 15 min. After washed with PBS for three times, the cells were permeabilized with 0.1% Triton X-100 (Biofroxx) in PBS for 10 min, then blocked with 5% goat serum (ThermoFisher) for 1 h. Coverslips were then incubated with appropriate primary antibodies overnight at 4 °C, and with Alexa-Fluor-conjugated secondary antibodies for 1 h. Coverslips were mounted on glass slides with Vectashield DAPI (Invitrogen) for nuclear staining. Fluorescence images were acquired using a confocal laser scanning microscope (FV1200, Olympus). The primary antibodies used were rabbit anti-insulin (15848-1-AP, Proteintech,1:200); mouse anti-proinsulin (ab243141, Abcam,1:100) and the secondary antibodies were Alexa Fluor^®^488 and Alexa Fluor^®^594 (Invitrogen, 1:200).

### Enzyme-linked immunosorbent assay (ELISA)

The levels of proinsuin (YM-1989A1, Youmeng Biotechnology Co., Ltd) and insulin (MS300, EZassay) in MIN6 cells supernatant were measured by ELISA according to the manufacturer’s instructions.

### Statistical analysis

Comparisons were performed using the Student’s t-test between two groups or one way ANOVA in multiple groups. The results were presented as the mean ± SD. *P* < 0.05 was considered statistically significant. Statistical analysis was performed using GraphPad Prism 9.0.

## Results

### Clinical characteristics of the patient and beta-cell function heterogeneity

**Fig. 1:**
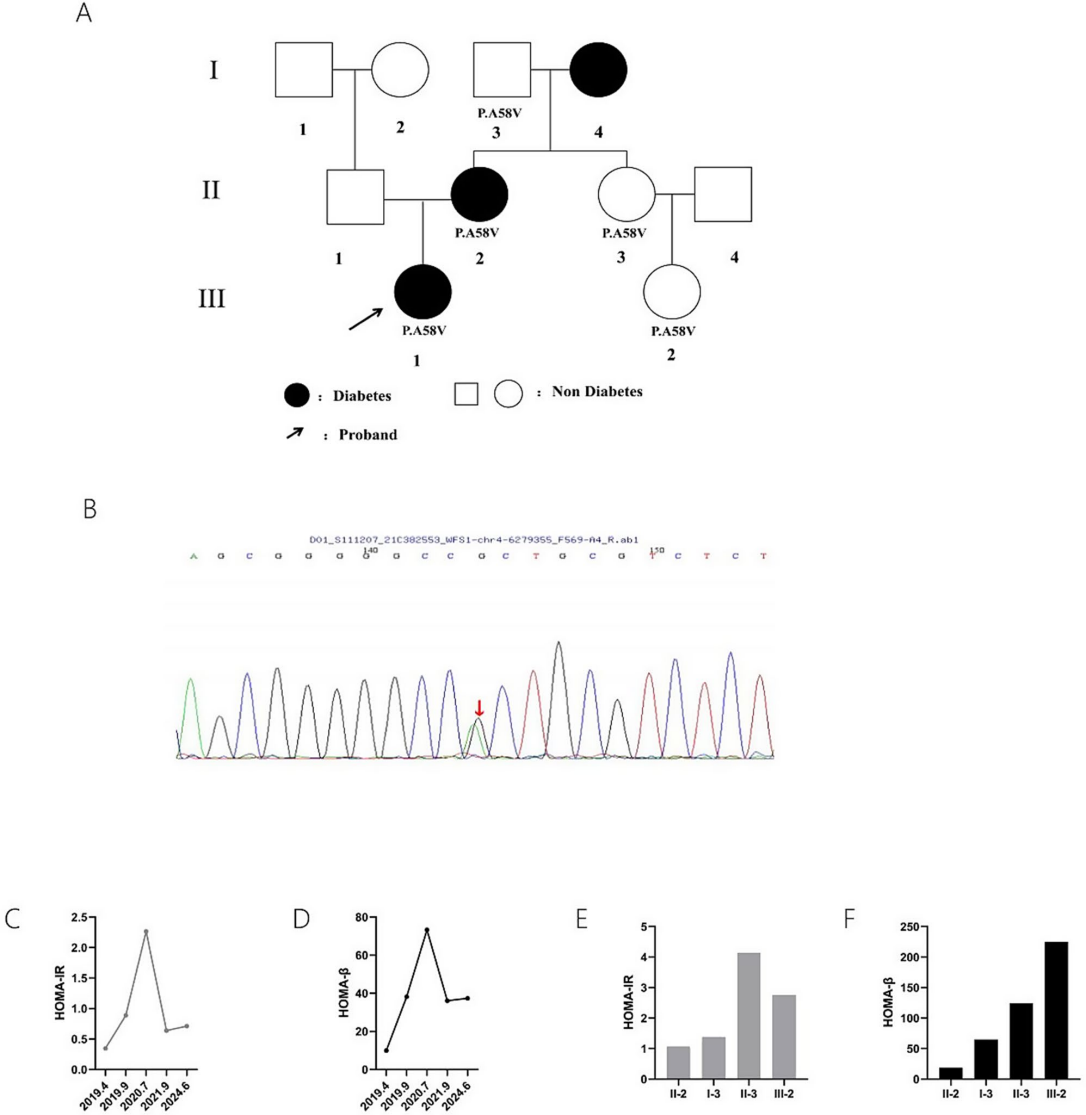
Family pedigree and beta-cell function of the patient and her family members. (**A**) Pedigree of the family. (**B**) Sanger sequencing peak map of the proband. (**C**,** D**) Five-year longitudinal follow-up of the proband’s beta-cell function. (**E**,** F**) Beta-cell function of the family members carrying the p.A58V mutation. I-3: grandfather of the proband; II-2: mother of the proband; II-3: maternal aunt of the proband; III-2: maternal cousin of the proband

The proband was a 14-year-old female with a documented history of diabetes spanning approximately one year. Laboratory data obtained from her initial hospitalization on April 1, 2019, indicated a random blood glucose level of 18.8 mmol/L and a positive urine ketone. Her glycated hemoglobin (HbA1c) was 13.1%, reflecting inadequate glycemic control and a ketosis onset tendency. The funduscopic examination and urinary system ultrasound were normal. Longitudinal monitoring from 2019 to 2024 included annual OGTT and HbA1c measurements were collated in Supplementary Tables 2, 4 and depicted in Fig. 1C, D, indicating initial impairment in beta-cell function and poor glycemic regulation, with subsequent improvement over the follow-up period. The patient tested negative for all islet autoantibodies, including glutamic acid decarboxylase antibody (GADA), insulinoma-associated antigen-2 antibody (IA2A), islet cell antibody (ICA), and insulin autoantibody (IAA). A familial history of diabetes was noted in her mother and maternal grandmother. Her mother has 2 years of diabetes history, taking glimepiride to control blood glucose; her maternal grandmother has 10 years of diabetes history, taking acarbose to maintain stable blood glucose level. The family’s pedigree was shown in Fig. 1A.

Intrafamilial phenotypic variation was evident in this study. Individuals harboring the same mutation underwent OGTT to assess beta-cell function (Supplementary Tables 3 and Fig. 1E, F). Despite the proband and her mother were diagnosed with DM, other carriers of the mutation within the family did not exhibit DM or any extra-pancreatic syndromes. Notably, a heterogeneity of beta-cell function within family members was observed. The HOMA-βvalue of the proband’s mother was 18.65, reflecting a reduced beta-cell function. The HOMA-IR value of the proband’s maternal aunt was 4.15, indicating more profound insulin resistance. The HOMA-β value of the proband’s maternal grandfather was 64.83, while the proband’s maternal cousin displayed a HOMA-β value of being 224.86 and a moderate HOMA-IR value of being 2.76, demonstrating normal beta-cell function. The proband’s beta-cell function was longitudinally monitored over a five-year period, revealing a dynamic trend of beta-cell function trajectory. The initial evaluation in 2019 indicated impaired beta-cell function. After resolution of glucotoxicity, a significant improvement in beta-cell function was observed in the second year of follow-up and has remained stable in subsequent follow-up visits.

### Mutation analysis of the WFS1 gene

**Fig. 2:**
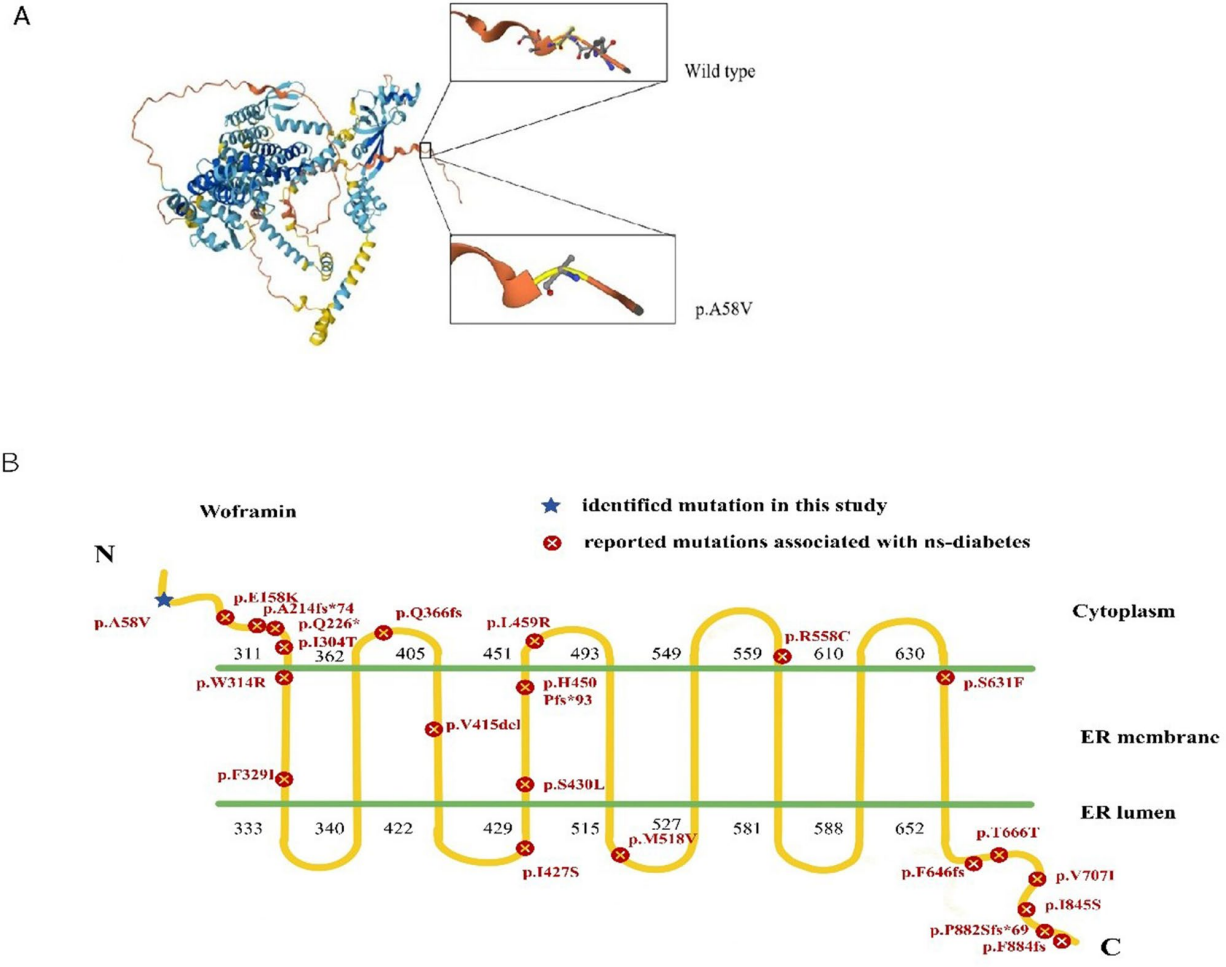
WFS1 mutation analysis. (**A**) Mutation induced structural changes in WFS1. (**B**) Mutation landscape of reported cases of ns-diabetes

A WFS1 gene mutation was identified in our study with heterozygous single nucleotide c.173 C > T substitution in exon 2. This nucleotide substitution result in an amino acid alteration of Arginine to Valine at position 58 in the protein product (p. A58V) (Fig. 2A).

The prediction results using multiple computational programs were shown in Table 1, demonstrating various results regarding the pathogenicity risk. We further explored the changes in the secondary or tertiary structure of the protein that may be caused by the p.A58V mutation, it shows a mild change which has subtle influence the whole structure (Fig. 2A).

**Table 1:** Pathogenicity assessment of WFS1 c.173 C > T (p.A58V)

Tools	Pathogenicity	Functional prediction scores/conservation scores
REVEL	Likely pathogenic	0.543
PolyPhen2	Possibly Damaging	0.516
MutationTaster	Polymorphism	1
GERP	Deleterious	3.24

To further elucidate the distribution of the mutation p.A58V, we illustrated a schematic depiction of the human Wolframin, the p.A58V mutation localized in the extracytoplasmic N-terminal domain of the Wolframin protein. Notably, we draw a overall landscape of reported mutations associated with the ns-WS (Fig. 2B), and summarized the clinical features (Supplementary Table 5). As shown in Fig. 2B, the ns-WS phenotype is involved in all the transmembrane regions of the Wolframin. The summary of the clinical features of the reported mutations reveals that patients exhibit diverse beta-cell function, and most were negative for islet autoantibodies and presented without diabetic ketoacidosis (DKA) at onset. However, the lack of comprehensive clinical data for the majority of patients limits our further exploration.

### WFS1 mutations activated ER stress

**Fig. 3:**
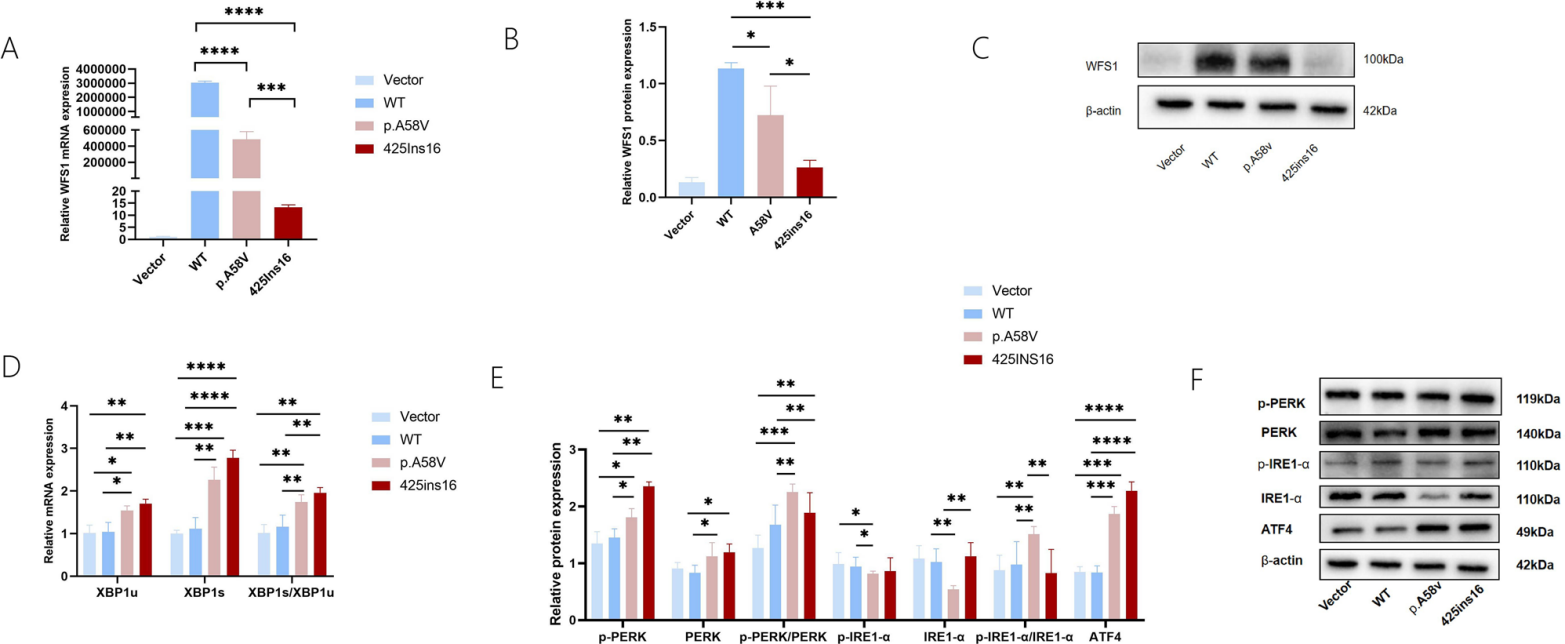
Effects of WFS1 mutations on ER stress. (**A**) Relative mRNA expression of WFS1. (**B**,** C**) Protein expression levels of Wolframin. (**D**) Quantitative RT-PCR for mRNA levels of XBP1u, XBP1s and the ratio of XBP1s/XBP1u. (**E**,** F**) Protein expression of ER stress related molecules. All reactions were performed in triplicate and the experiment was repeated 3 times. Data are shown as mean ± SD, *p* < 0.05, significant. Vector, pcDNA3.1; WT, wild type. *p < 0.05; **p < 0.01; ***p < 0.001; ****p < 0.0001

Firstly, we evaluated Wolframin protein expression in MIN6 cells transfected with WFS1 mutation-carrying plasmids. Both the p.A58V and 425ins16 mutations showed significantly reduced Wolframin levels, with the 425ins16 mutation demonstrates nearly absence of Wolframin (Fig. 3A, B, C).

Disruption in ER homeostasis triggers the unfolded protein response (UPR), a compensatory mechanism to mitigate ER stress. AS the activation of ER stress composed the key mechanism of beta cell dysfunction, subsequently, we performed functional analysis concerning the markers of three primary pathways of UPR [12]. RT- qPCR results confirmed that the levels of XBP1u, XBP1s, and the ratio of XBP1s/XBP1u were elevated in both p.A58V and 425ins16 (Fig. 3D). The protein expression of UPR showed significant differences between WT and p.A58V, and were higher in the 425ins16, indicating a worse clinical phenotype (Fig. 3E, F).

### WFS1 deficiency impedes proinsulin processing

**Fig. 4:**
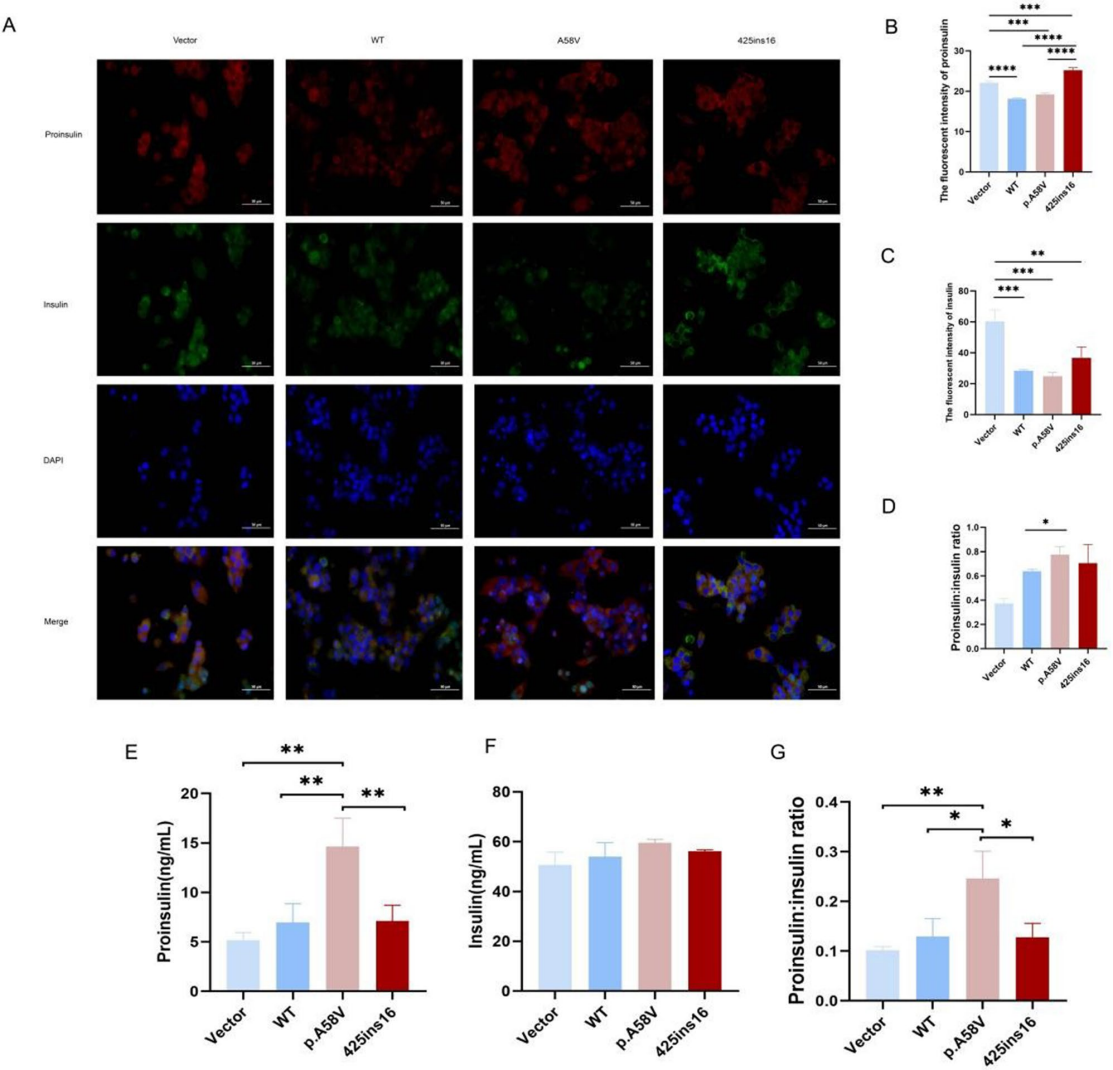
Effects of WFS1 mutations on insulin processing. (**A**) Confocal microscopy of proinsulin and insulin in WT and WFS1 mutation (p.A58V, 425ins16). MIN6 cells were immunostained with anti-proinsulin and anti-insulin primary antibodies, followed by Alexa Fluor-conjugated secondary antibodies. Scale bar, 50 μm. (**B**) The fluorescence intensities of proinsulin. (**C**) The fluorescence intensities of insulin. (**D**) The ratio of fluorescence intensity of proinsulin to insulin. (**E**) The level of proinsulin. (**F**) The level of insulin. (**G**) The ratio of proinsulin to insulin. *n* = 3 independent experiments, *n* = 24 independent images quantified. All the data are presented as mean ± SD. *p* < 0.05, significant, using One-way analysis of variance (ANOVA). Vector, pcDNA3.1; WT, wild type. *p < 0.05; **p < 0.01; ***p < 0.001; ****p < 0.0001

By using immunostaining analyses, we observed a notable discrepancy in proinsulin distribution between WT and WFS1 mutations (Fig. 4A). The proinsulin levels in the mutant WFS1 cells were increased compared to WT (Fig. 4B). The insulin levels decreased in the p.A58V mutation (Fig. 4C). Importantly, the p.A58V mutation exhibited an elevated proinsulin to insulin ratio relative to WT (Fig. 4D). ELISA data also showed a significantly higher proinsulin-to-insulin ratio in the p.A58V mutant cells, which was consistent with the result of the immunostaining findings (Fig. 4E, F, G). This suggests that WFS1 mutation results in abnormal proinsulin accumulation in the ER, consequently hindering the proinsulin processing and insulin secretion.

## Discussion

In this study, we identified a missense WFS1 mutation p.A58V which is associated with non-syndromic DM. We conducted a longitudinal observation of the proband, revealing dynamic trend of beta-cell function over time. Meanwhile, a comparison of beta-cell function among family members highlighted its heterogeneity. Further in vitro functional analyses indicated that the WFS1 mutation leads to DM phenotype predominantly through the activation of ER stress and the impairment of insulin processing, providing a comprehensive understanding of this phenotype.

Classical WS is recognized as a recessive inherited disease primarily characterized by the combination of juvenile-onset DM and OA [13]. Interestingly, the current case highlights an individual without typical WS features, suggesting non-syndromic DM represents a rare phenotype of WS. Previous reports of non-syndromic DM in WS are sporadic. This phenotype was firstly reported in the Lebanon population in 2008, this phenotype accounted for 12.1% of probands in Lebanese consanguineous families [14]. Subsequently in 2013, Bonnycastle [15] et al. identified a nonsynonymous mutation in WFS1 causing dominantly inherited, adult-onset, non-syndromic DM. Additionally, homozygosity for a rare missense variant (c.1672 C > T, p.R558C) in the WFS1 gene was reported in Ashkenazi Jews ancestry, with the same variant observed in Southern Indian [4, 16]. In this latter instance, a 17-year-old male presented with young-onset diabetes without OA, initially misdiagnosed as T2DM due to his BMI was 30.5 kg/m^2^ and absence of autoimmune markers. Our previous research suggests a potential prevalence of non-syndromic DM within the Chinese population, with identifying four cases of WFS1-related non-syndromic DM among 82 Chinese patients clinically diagnosed as T1DM but lacking autoimmune markers [17]. Similarly, in a separate Chinese cohort of 105 pancreatic autoantibody-negative patients, three cases of non-syndromic DM due to recessive WFS1 mutation were identified, representing an incidence of 0.94% [18]. These findings contribute to broadening the genetic and clinical phenotypic associated with WS.

To further elucidate the genetic and clinical spectrum of WFS1-related DM, we made a summary of the clinical features and the reported mutations phenotyped as isolated DM (supplementary Table 5, Fig. 1B). There were 28 patients identified as isolated DM, a considerable proportion of the reported WFS1 mutations associated with milder diabetic phenotypes were found to be in a heterozygous state. This indicate that a single functional copy of WFS1 may maintain a certain level of β-cell function, while homozygous or compound heterozygous mutations lead to complete loss of β-cell function. This heterozygous effect could explain the variability in the age of onset and the severity of glycemic control observed among different patients with WFS1-related diabetes. However, most of the reported cases lack comprehensive data collection. Therefore, it is important to collect the detailed clinical information to enhance understanding of phenotype. Loss of function in the WFS1 gene was the primary cause of WS. To date, over 200 mutations have been documented in WS patients, including pathogenic mutations such as frameshift deletions, missense changes and insertions within WFS1 gene [19]. A significant concentration of these mutations is concentrated in exon 8, particularly in regions that encodes the C-terminus and transmembrane segments. Loss of function of these domains is thought to activate ER stress, which are key mechanisms underlying the symptoms in WS [20]. The WFS1 mutation c.173 C > T (p.A58V) was previously identified in a compound heterozygous state with c.1558 C> T (p.Q520X) mutation in a Spanish female patient, who developed DM at age 8 and OA at age 14. The c.1558 C > T variant leads to a truncated protein at residue 520, replacing a glutamine codon (CAG) with a stop codon (UAG). It’s hypothesized that the p.A58V mutation probably represents a minor alternation of Wolframin, contributing to a milder phenotype [11]. As a result, the main pathogenic effect may be caused by the c.1558 C > T variant.

Our study found that activation of ER stress and insulin synthesis dysfunction are the main mechanisms underlying the isolated diabetes phenotype of Wolfram syndrome. The ER plays a crucial role in the regulation of UPR and maintenance of the Ca^2+^ storage, emerging evidence suggests that homeostatic alterations within the ER are intricately linked to beta-cell dysfunction, which are pivotal in the onset and progression of DM [21]. In our study, we demonstrated the upregulation of the ER stress target genes, including IRE1, the PERK, the XBP1 and the ATF4, resulting in activation of UPR. Functional analysis revealed that, unlike the profound effect of 425ins16, the p.A58V mutation exhibited a mildly higher effect compared to the WT, which could partly explain the relatively mild phenotype.

Insulin synthesis and processing dysfunction is another major mechanism of this phenotype, as our mutation is located at the N-terminal of the Wolframin, a site more prone to disrupting insulin synthesis. Previous researches have elucidated that the WFS1 gene regulates pathways maintaining beta-cell functionality [22]. Loss of function of WFS1 is implicated in a concomitant impairment in stimulus-secretion. Our analysis revealed impaired insulin processing, represented by proinsulin accumulation. The mutation of interest is situated in exon 2 near the N-terminus, this is parallel with a recently published literature which indicates that the WFS1 gene functions in the transfer of insulin processing from the ER to the Golgi complex [23], The pathogenic mutations in the N-terminus would undermine the communication between the Golgi complex and the coat protein complex II (COPII), thereby hindering proinsulin processing as well as secretion, results in an accumulation of proinsulin [23].

Previous studies have demonstrated that beta-cell function may serve as a valuable biomarker for the progression of WFS1-related DM [24]. Our study observed the longitudinal changes in beta-cell function over five years, as well as the heterogeneity of beta-cell function among carriers. With the improvement of glycemic control, the proband’s dynamic trend of beta-cell function remains stable over five years, with no significant decline observed. Meanwhile, there is intra-family heterogeneity of beta-cell function. The proband’s mother exhibited impaired beta-cell function, while the proband’s maternal aunt showed more profound insulin resistance. The proband’s maternal grandfather had a decline in beta-cell function, which may be related to the aging process. In contrast, the proband’s maternal cousin displayed normal beta-cell function. Reported studies associated with ns-WS were presented with incomplete clinical information and a lack of longitudinal follow-up, our study is the longest follow-up study to date on beta-cell function in patients with WFS1 mutations.

Longitudinal follow-up of islet function is also performed to differentiate from type 1 diabetes, as islet function in type 1 diabetes often declines rapidly. In the clinical, both T1DM and WFS1-related DM manifest during childhood or adolescence. Distinguishing between these two conditions necessitates a multifaceted approach, encompassing genetic testing and comprehensive clinical assessments. WFS1-related DM typically emerges with a less severe diabetic phenotype initially. Unlike T1DM, an autoimmune disorder characterized by specific autoantibodies such as GADA, IA-2A, and IAA, WFS1-related DM generally does not exhibit these markers. Furthermore, beta-cell dysfunction in WFS1-related DM may be observed prematurely, in the absence of the characteristic autoimmune-mediated beta-cell destruction associated with T1DM. Familial history can also be indicative of WFS1-related DM. The most conclusive diagnostic differentiation is achieved through genetic testing, identifying mutations within the WFS1 gene. Patients with WFS1-related DM may have a different response to insulin or oral hypoglycemic agents compared to those with T1DM.

While our study provides valuable insights into the pathogenesis of non-syndromic diabetes induced by the WFS1 p.A58V missense mutation, several limitations should be acknowledged. Primarily, our conclusions regarding the phenotypic heterogeneity of the p.A58V mutation are drawn from a single family, while informative, larger cohort studies are needed to fully understand the range of beta-cell function and potential extra-pancreatic manifestations associated with this specific mutation. Furthermore, the functional characterization of the p.A58V mutation was primarily conducted using MIN6 cells. Expanding these investigations to patient-derived beta-cells or other relevant cell types would offer further validation and a more comprehensive understanding of the mutation’s functional consequences.

In the present study, we identified a rare WFS1 mutation, p.A58V, that is associated with non-syndromic DM, diverging from the full spectrum of clinical manifestations typically linked to Wolfram syndrome. This investigation provides the first functional mutational report in a pedigree with non-syndromic diabetes. Our results contribute a critical piece to the compendium of WFS1 mutations, expanding the current understanding of the WFS1 gene’s role in DM and enriching the dialogue on genotype-phenotype correlations.

## Supplementary Information

Below is the link to the electronic supplementary material.

Supplementary Material 1

Supplementary Material 2

## Data Availability

The data that support the findings of this study are available from the corresponding author upon reasonable request.
