# Supplementary material for: WFS1-related isolated diabetes induced by a WFS1 missense mutation: focus on the isolated diabetes phenotype
Source: Orphanet J Rare Dis. 2026 Mar 27;21:180. doi: 10.1186/s13023-026-04291-9 (PMC13147663; doi:10.1186/s13023-026-04291-9)
Supplement: Supplementary file 1 — Supplementary Material 1 [file 13023_2026_4291_MOESM1_ESM.docx]

**Supplementary materials**

**Table 1: The qPCR primer sequences used in this study.**

| **Gene name** | **Forward Primer Sequence** | **Reverse Primer Sequence** |
| --- | --- | --- |
| XBP1u | GACAGAGAGTCAAACTAACGTGG | GTCCAGCAGGCAAGAAGGT |
| XBP1s | ACACGCTTGGGAATGGACAC | CCATGGGAAGATGTTCTGGG |
| β-actin | GGCTGTATTCCCCTCCATCG | CCAGTTGGTAACAATGCCATGT |
| WFS1 | GCGTGACTGACATCGACAAC | GTAGCGGTCGAACTTCTTGA |

**Table 2:** **The longitudinal follow-up of the proband’s beta-cell function.**

| Follow-up time |  | 0min | 60min | 120min |
| --- | --- | --- | --- | --- |
| 2019.4 | Glucose(mmol/l) | 6.08 | 14.97 | 18.78 |
|  | Insulin(pmol/l) | 7.7 | 47.03 | 50.48 |
|  | C-peptide(pmol/l) | 157.9 | 515.4 | 715.7 |
| 2019.9 | Glucose(mmol/l) | 5.43 | 11.01 | 12.13 |
|  | Insulin(pmol/l) | 22.1 | 122.2 | 182.6 |
|  | C-peptide（pmol/l） | 376.1 | 916 | 1610 |
| 2020.7 | Glucose(mmol/l) | 5.87 | 11.58 | 13.84 |
|  | Insulin(pmol/l) | 52.1 | 177.2 | 170.2 |
|  | C-peptide(pmol/l) | 489.7 | 1065 | 1572 |
| 2021.9 | Glucose(mmol/l) | 5.07 | 12.56 | 14.75 |
|  | Insulin(pmol/l) | 17 | 141.4 | 117.2 |
|  | C-peptide(pmol/l) | 354 | 995.1 | 1208 |
| 2024.6 | Glucose(mmol/l) | 5.16 | 12.79 | 13.4 |
|  | Insulin(pmol/l) | 18.6 | 142.8 | 107.9 |
|  | C-peptide(pmol/l) | 433.8 | 1134 | 1554 |

**Table 3: The beta-cell function of the family members who carry the same WFS1 mutation.**

| Family members |  | 0min | 60min | 120min |
| --- | --- | --- | --- | --- |
| II-2 | Glucose(mmol/l) | 7.11 | 12.41 | 12.11 |
|  | Insulin(pmol/l) | 20.2 | 131.7 | 195.5 |
|  | C-peptide(pmol/l) | 424.4 | 1167 | 2141 |
| II-3 | Glucose(mmol/l) | 6.00 | 10.16 | 6.27 |
|  | Insulin(pmol/l) | 93.3 | 1039 | 884.7 |
|  | C-peptide(pmol/l) | 996.4 | 4655 | 4279 |
| III-2 | Glucose(mmol/l) | 4.68 | 6.06 | 3.55 |
|  | Insulin(pmol/l) | 79.6 | 858.1 | 370 |
|  | C-peptide(pmol/l) | 615.3 | 3648 | 2542 |
| I-3 | Glucose(mmol/l) | 5.31 | 7.73 | 6.12 |
|  | Insulin(pmol/l) | 35.2 | 436 | 349 |
|  | C-peptide(pmol/l) | 550.1 | 2966 | 2983 |

**Table 4: The longitudinal follow-up of the proband’s HbA1c**

| Follow-up time | HbA1c(%) |
| --- | --- |
| 2019.4 | 13.1 |
| 2019.9 | 5.9 |
| 2020.7 | 6.4 |
| 2021.9 | 5.7 |
| 2024.9 | 5.5 |

**Table 5: Summary of the clinical features and the reported mutations phenotyped as isolated DM.**

| **Patient** | **Mutation** | **Age at onset of diabetes** | **Sex** | **BMI (kg/m^2^)** | **HbA1c at onset of diabetes (%)** | **Islet autoimmune antibodies** | **Fasting plasma glucose**  **(mmol/L)** | **Fasting insulin**  **(pmol/L)** | **Fasting C-peptide**  **(pmol/L)** | **DKA at onset** | **Ref.** |
| --- | --- | --- | --- | --- | --- | --- | --- | --- | --- | --- | --- |
| 1 | V707I+F884fs951X | 7 | — | — | — | — | — | — | — | — | (1) |
| 2 | V707I+F884fs951X | 5 | — | — | — | — | — | — | — | — |  |
| 3 | V707I+F884fs951X | 5 | — | — | — | — | — | — | — | — |  |
| 4 | V707I+F884fs951X | 3 | — | — | — | — | — | — | — | — |  |
| 5 | V707I+F884fs951X | 7 | — | — | — | — | — | — | — | — |  |
| 6 | V707I+F884fs951X | 5 | — | — | — | — | — | — | — | — |  |
| 7 | V707I+F884fs951X | 8 | — | — | — | — | — | — | — | — |  |
| 8 | V707I+F884fs951X | 4 | — | — | — | — | — | — | — | — |  |
| 9 | V707I+F884fs951X | 7 | — | — | — | — | — | — | — | — |  |
| 10 | V707I+F884fs951X/F646fs708X | 6 | — | — | — | — | — | — | — | — |  |
| 11 | p.W314R | 51 | F | 23 | — | Negative | 7.0 | 41.67 | 640 | No | (2) |
| 12 | p.W314R | — | M | 21.5 | — | Negative | 6.0 | 83.34 | 760 | No |  |
| 13 | p.R558C | 14 | — | — | — | Negative | >10 | — | — | No | (3) |
| 14 | p.Q366fs/p.L459R | 9 | — | — | 15.6 | Negative | — | — | 170.16 | — | (4) |
| 15 | p.E158K/p.F329I | 18 | — | — | — | Negative | — | — | 111.7 | — |  |
| 16 | p.E158K/p.F329I | 22 | — | — | — | Negative | — | — | — | — |  |
| 17 | p.S631F | 5 | — | — | — | Negative | — | — | 69.93 | — |  |
| 18 | p.V415del/p.I845S | 5.7 | M | 17.8 | 6.3 | Negative | 6.7 | 42.36 | 757 | No | (5) |
| 19 | p.Trp666Ter/p.P882Sfs*69 | 3.3 | M | 16.9 | 7.2 | Negative | 8.2 | — | 827 | — |  |
| 20 | p.Trp666Ter/p.P882Sfs*69 | 7 | F | 17.2 | 10.9 | Negative | 7.9 | — | 970 | — |  |
| 21 | p.H450Pfs*93 | 68 | F | 27.34 | — | — | — | — | — | — | (6) |
| 22 | p.A214fs*74/p.F329I | 25 | F | 18.6 | 5.6 | Negative | 6.94 | — | 445.41 | No | (7) |
| 23 | p.I427S/p.I304T | 13 | F | 20.06 | 13.2 | Negative | 11.2 | — | 360.57 | No |  |
| 24 | p.S430L | 25 | M | 28.2 | 11.5 | Negative | 13.27 | — | 430.26 | No |  |
| 25 | p.M518V | 25 | F | 23.18 | 6.4 | Negative | 9.64 | — | 890.82 | No |  |
| 26 | p.Q226* | 25 | M | 22.8 | 6.4 | Negative | 7.36 | — | 672.66 | No |  |

“—”, not provided. F, female; M, male. BMI, body mass index. HbA1c, glycated hemoglobin. DKA, diabetic ketoacidosis

1. **Zalloua PA, Azar ST, Deelépine M, Makhoul NJ, Blanc H, Sanyoura M, Lavergne A, Stankov K, Lemainque A, Baz P, Julier C.** WFS1 mutations are frequent monogenic causes of juvenile-onset diabetes mellitus in Lebanon. *Human Molecular Genetics* 2008;17(24):4012–4021.

2. **Bonnycastle LL, Chines PS, Hara T, Huyghe JR, Swift AJ, Heikinheimo P, Mahadevan J, Peltonen S, Huopio H, Nuutila P, Narisu N, Goldfeder RL, Stitzel ML, Lu S, Boehnke M, Urano F, Collins FS, Laakso M.** Autosomal dominant diabetes arising from a wolfram syndrome 1 mutation. *Diabetes* 2013;62(11):3943–3950.

3. **Bansal V, Boehm BO, Darvasi A.** Identification of a missense variant in the WFS1 gene that causes a mild form of Wolfram syndrome and is associated with risk for type 2 diabetes in Ashkenazi Jewish individuals. *Diabetologia* 2018;61(10):2180–2188.

4. **Li M, Wang S, Xu K, Chen Y, Fu Q, Gu Y, Shi Y, Zhang M, Sun M, Chen H, Han X, Li Y, Tang Z, Cai L, Li Z, Shi Y, Yang T, Polychronakos C.** High Prevalence of a Monogenic Cause in Han Chinese Diagnosed With Type 1 Diabetes, Partly Driven by Nonsyndromic Recessive WFS1 Mutations. *Diabetes* 2020;69(1):121–126.

5. **Zhu M, Li Y, Dong G, Chen X, Huang K, Wu W, Dai Y, Zhang L, Lin H, Wang S, Polychronakos C, Fu J.** Prevalence and phenotypic features of diabetes due to recessive, non-syndromic WFS1 mutations. *European journal of endocrinology* 2021;186(2):163–170.

6. **Ding Y, Li Z, Zhang Q, Li N, Chang G, Wang Y, Li X, Li J, Li Q, Yao R-E, Li X, Wang X.** Complex clinical manifestations and new insights in RNA sequencing of children with diabetes and WFS1 variants. *Frontiers In Endocrinology* 2023;14:1066320.

7. **Wu L, Zhang J, Li D, Zhang Z, Ni Q, Han R, Ye L, Zhang Y, Hong J, Wang W, Ning G, Gu W.** Novel WFS1 variants are associated with different diabetes phenotypes. *Frontiers In Genetics* 2024;15:1433060.
