# Supplementary figures and images for: WFS1-related isolated diabetes induced by a WFS1 missense mutation: focus on the isolated diabetes phenotype
Source: Orphanet J Rare Dis. 2026 Mar 27;21:180. doi: 10.1186/s13023-026-04291-9 (PMC13147663; doi:10.1186/s13023-026-04291-9)

（1）WFS1


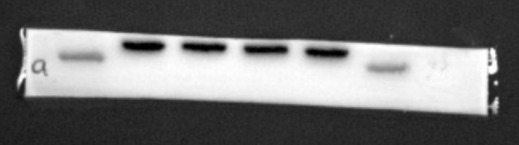


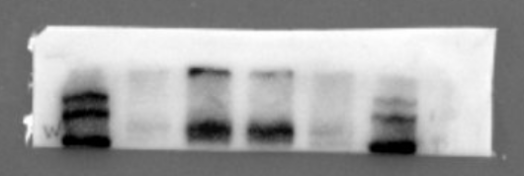


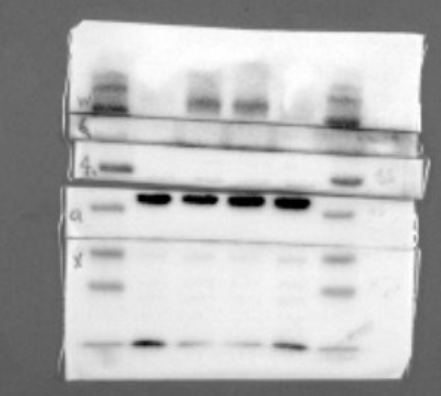


1. PERK；pPERK

PERK


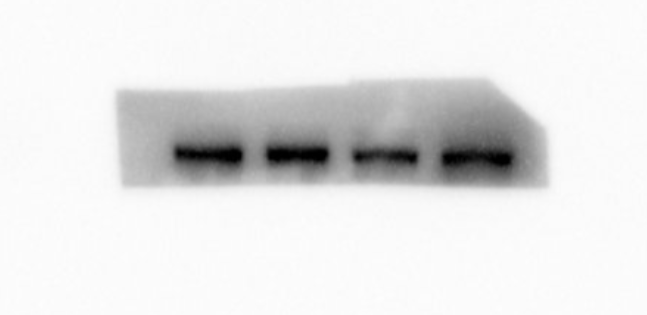


β-actin


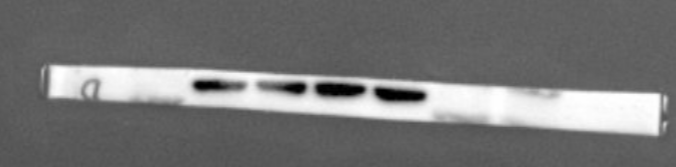


pPERK


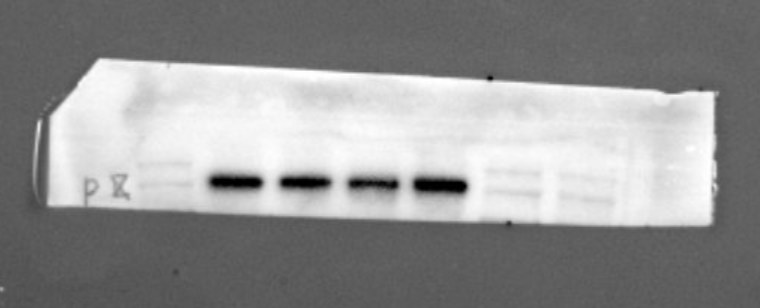


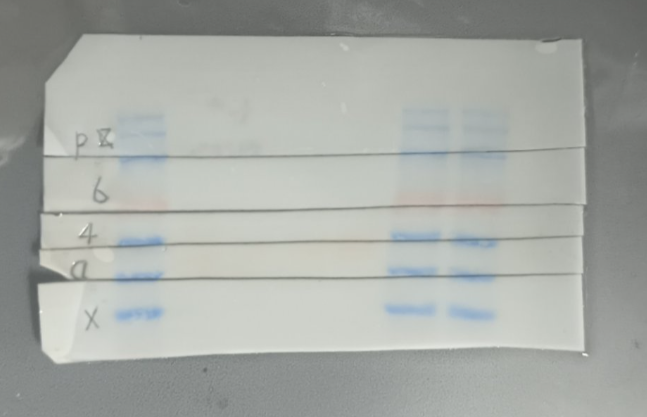


1. P-IRE1-α


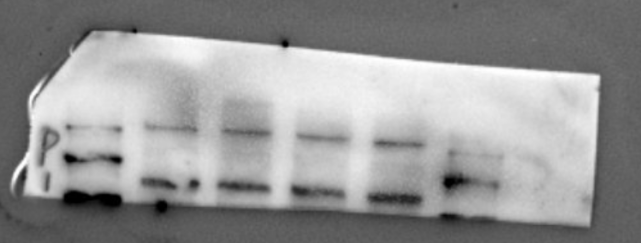


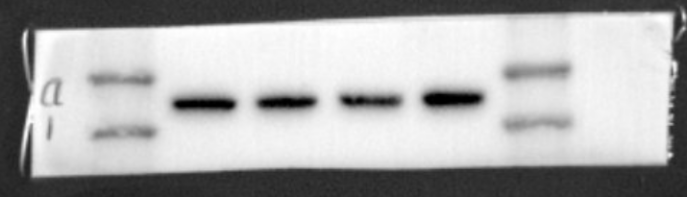


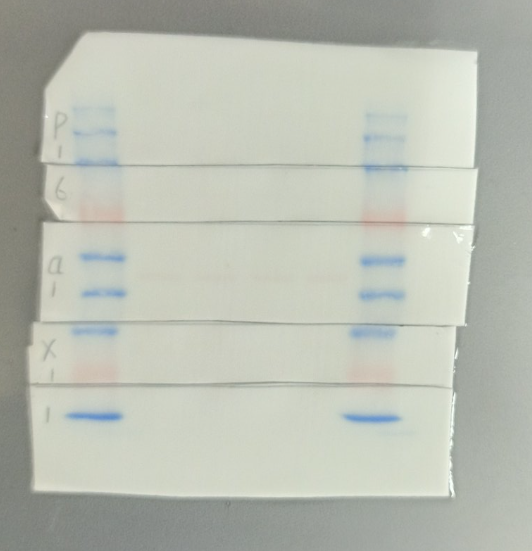


（4）IRE1-α；


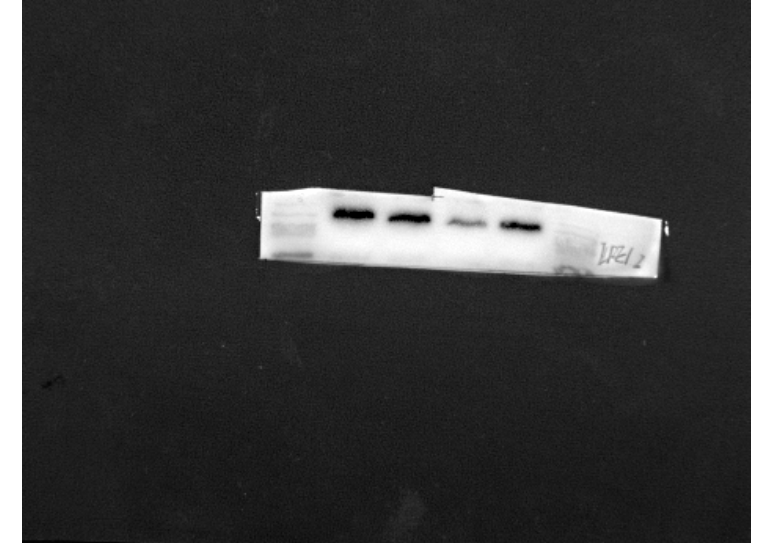


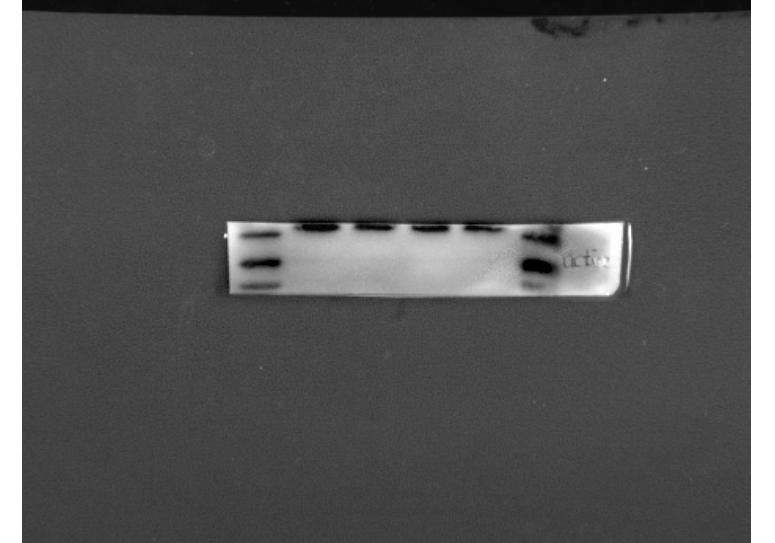


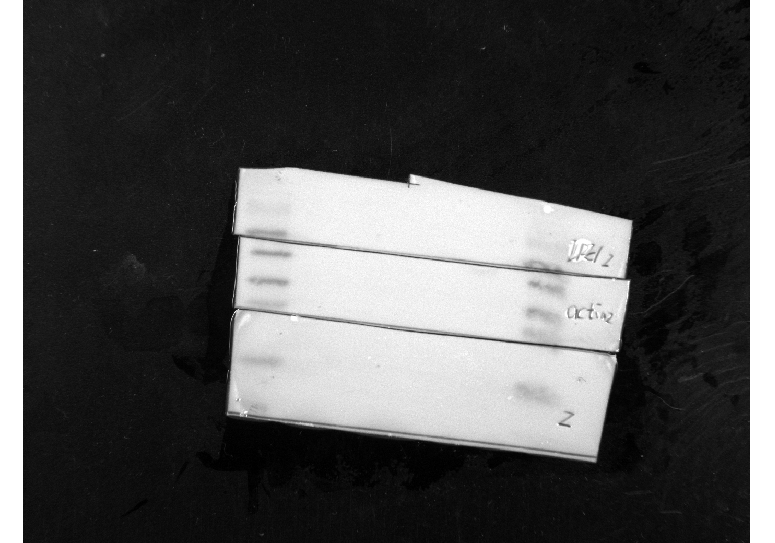


1. ATF4


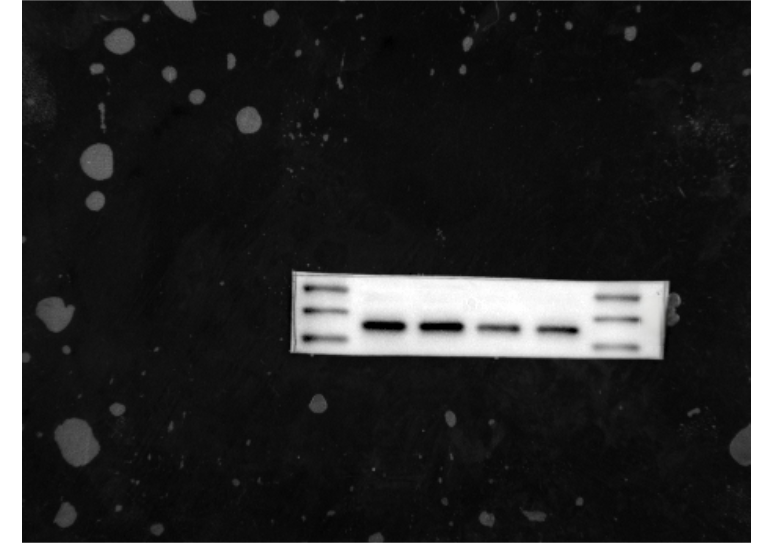


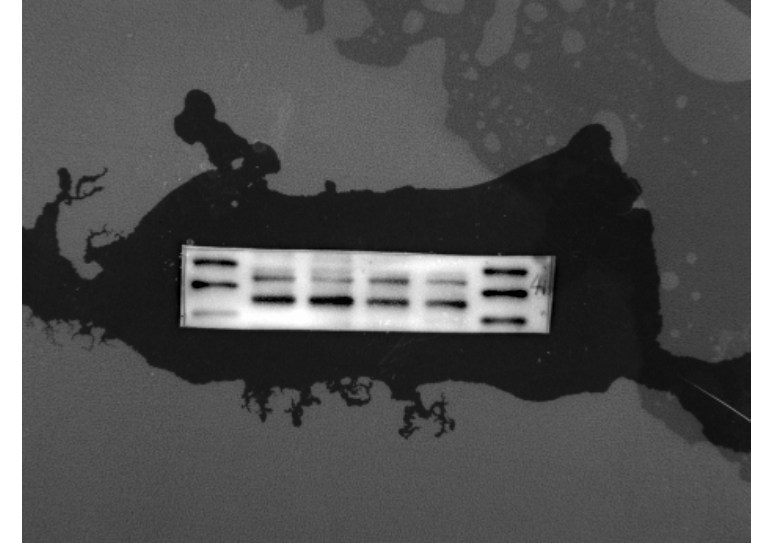


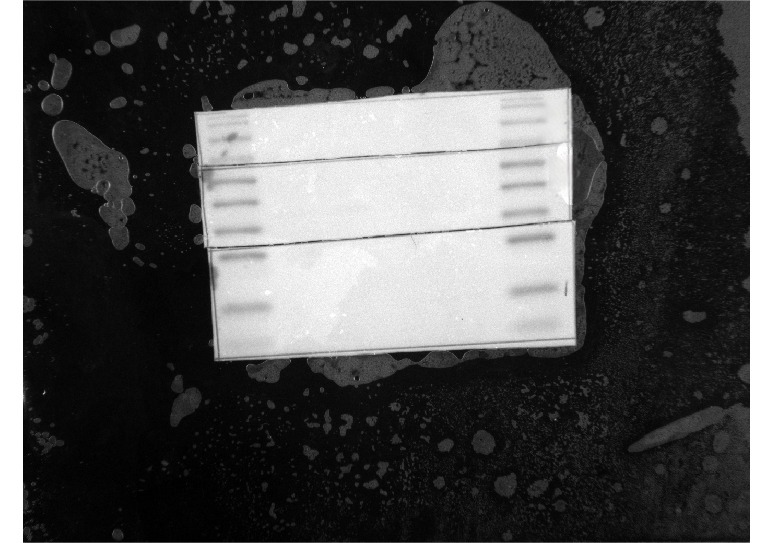

Supplement: Supplementary file 2 — Supplementary Material 2 [file 13023_2026_4291_MOESM2_ESM.docx]
